# Efficacy of movement control exercises versus general exercises on recurrent sub-acute nonspecific low back pain in a sub-group of patients with movement control dysfunction. protocol of a randomized controlled trial

**DOI:** 10.1186/1471-2474-13-55

**Published:** 2012-04-11

**Authors:** Vesa Lehtola, Hannu Luomajoki, Ville Leinonen, Sean Gibbons, Olavi Airaksinen

**Affiliations:** 1 Department of Physical and Rehabilitation Medicine, Institute of Clinical Medicine, University of Eastern Finland, Kuopio, Finland; 2 Zürich University of Applied Sciences ZHAW, Institut for Physiotherapy, Winterthur, Switzerland; 3 Neurosurgery of NeuroCenter, Kuopio University Hospital, Kuopio, Finland; 4 SMARTERehab, Neuromuscular Rehabilitation Institute, Newfoundland, Canada; 5 Department of Physical and Rehabilitation Medicine, University of Eastern Finland, Institute of Clinical Medicine and Kuopio University Hospital, Kuopio, Finland

## Abstract

**Background:**

Practice guidelines recommend various types of exercise for chronic back pain but there have been few head-to-head comparisons of these interventions. General exercise seems to be an effective option for management of chronic low back pain (LBP) but very little is known about the management of a sub-acute LBP within sub-groups.

Recent research has developed clinical tests to identify a subgroup of patients with chronic non-specific LBP who have movement control dysfunction (MD).

**Method/Design:**

We are conducting a randomized controlled trial (RCT) to compare the effects of general exercise and specific movement control exercise (SMCE) on disability and function in patients with MD within recurrent sub-acute LBP. The main outcome measure is the Roland Morris Disability Questionnaire.

**Discussion:**

European clinical guideline for management of chronic LBP recommends that more research is required to develop tools to improve the classification and identification of specific clinical sub-groups of chronic LBP patients. Good quality RCTs are then needed to determine the effectiveness of specific interventions aimed at these specific target groups. This RCT aims to test the hypothesis whether patients within a sub-group of MD benefit more through a specific individually tailored movement control exercise program than through general exercises.

## Background

### LBP epidemiology

LBP is a common, costly problem, often associated with high recurrence rates and equivocal management efficacy [1,2]. LBP remains the primary cause of absenteeism and disability in every industrialized society [3].

Patients who develop chronic LBP (pain and disability persisting for more than 3 months) use more than 80% of all health care for back pain [3].

A recent systematic review of the prognosis of acute LPB showed that the view of spontaneous healing is inaccurate. Pain and disability are typically ongoing, and recurrences are common. Up to 70% of those who initially improve, experience repeated fluctuating pain episodes [4]. Thus, effective treatments for patients whose pain and disability persist beyond the acute phase are needed.

We are interested in the sub-acute phase, which is the transition period from acute (duration less than 6 weeks) to chronic (duration over 3 months) LBP.

The European Guidelines for Management of Chronic Non-Specific LBP recommend supervised exercise therapy as a first-line treatment for chronic LBP [5]. Exercise therapy appears to be slightly effective in decreasing pain and improving function in adults with chronic LBP, particularly in healthcare populations. In sub-acute LBP there is evidence that a graded activity program improves absenteeism outcomes, though evidence for other types of exercise is unclear [6,7].

A cost effectiveness study compared the costs and benefits of a graded activity intervention

To usual care for sick-listed workers with nonspecific sub-acute LBP. After a 3-year follow-up it could be concluded that the graded activity intervention for non-specific LBP is a cost-beneficial return-to-work intervention [8]. An RCT compared the effects of stabilizing training and manual therapy in sub-acute and chronic LBP. The results did not indicate any clear short-term differences between the groups in the accessed outcome measures. In the long term, however, stabilizing training seemed to be more effective than manual treatment in terms of improvement of individuals and the reduced need for recurrent treatment periods [9]. Another RCT indicated that in participants with sub-acute LBP, physiotherapist-directed exercise and advice were each slightly more effective than placebo at 6 weeks. The effect was greatest when the interventions were combined. At 12 months, the only effect that persisted was a small effect on participant-reported function [10].

A few studies have tried to find out the prognostic factors and the transition from acute or sub-acute LBP to chronic pain. A prospective cohort study of patients with episodes of acute or sub-acute LBP, seeking physical therapy in primary care, with follow-up at weeks 2, 4, 8, and 12 strongly revealed pain-related items to be essential factors in the development of chronic and long-term disability in primary care physical therapy. Health status at 8 weeks seemed crucial in developing chronic pain [11]. A Dutch cluster RCT provided no evidence that general practitioners should adopt a new treatment strategy aimed at psychosocial prognostic factors in patients with sub-acute LBP [12].

A prospective cohort study demonstrated that physical parameters did not have a prognostic value with regard to outcome of treatment. Furthermore, the data confirmed that

Patients' subjective estimation of pain and disability already displays a prognostic value for therapy outcome that cannot be increased significantly by the assessment of physical parameters [13].

Chronic non-specific LBP has been studied with many exercise interventions. The types of exercise programs for chronic LBP vary widely e.g. land-based exercise versus exercise in water, individual exercise versus group exercise, isolated trunk exercise versus whole body exercise. Unfortunately there is little or no evidence to help clinicians select the most effective type of exercise for an individual patient. This absence of evidence means that care is likely to be sub-optimal. While some trials of exercise have reported large, durable and clinically important effects [14], others have not [15]. The types of exercise programs, and patient presentations for chronic LBP vary widely so it is unlikely that all programs are equally effective for all patients[6].

### General exercise and standard therapy

There is not a 'standard therapy' for any type (acute, sub-acute, chronic) of LBP that is agreed upon to use as a comparison in clinical trials. Exercise therapy is recommended by various guidelines [5,16], but it is not clear which type of exercises are best.

The use of general exercise is problematic because there are so many types of exercise that may be considered under this umbrella term [17].

One study [18] compared general exercise, motor control exercise and manual therapy in treating chronic LBP. Cardiovascular aerobic and main muscle group strengthening exercises were considered general exercise. Muscle strengthening exercises were conducted with weights. Koumantakis et al. (2005) defined general exercise as targeting abdominal and paraspinal muscles without the involvement of the deep muscles activation [19]. A systematic review by Keating et al. (2006) referred to general exercise as muscle strengthening, coordination and aerobic fitness improving exercises [20]. The same approach had been used by Dvorak et al. (2001) [21]. Classic trunk exercises performed in physical therapy activate the abdominal and paraspinal muscles as a whole and at a relatively high contraction level [22,23].

As a conclusion the term general exercises can involve strengthening exercises for all the main muscle groups with or without the addition of weights. In addition, the term can involve exercises improving coordination, stretching and aerobic fitness training.

According to the literature, general exercises seem to be an effective treatment for non-specific LBP in physiotherapy. The benefits include: pain reduction, improved working ability, increased function, reduced depression and reduced fear of pain. However, the results are comparable to those with specific exercise, especially in the longer term. The short term benefits for specific training methods are potentially even more effective in reducing pain [18,24-30].

### Sub-classification of low back pain patients

The heterogeneity of the patients with non-specific LBP has been problematic. The sub-grouping these patients was declared to be one of the main focuses for future research a decade ago. Emphasis is to view LBP as a multi-factorial biopsychosocial pain syndrome [31].

A systematic review with a meta-analysis has been published to determine the integration of sub-classification strategies with matched interventions in RCTs evaluating manual therapy treatment and exercise therapy for chronic non-specific LBP. Only 5 of 68 studies (7.4%) sub-classified patients beyond applying general inclusion and exclusion criteria. In the few studies where classification and matched interventions have been used, meta-analysis showed a statistical difference in favour of the classification-based intervention for reductions in pain (both for short-term and long-term) and disability. Effect sizes ranged from moderate (0.43) for short term to minimal (0.14) for long term. The authors concluded that a better integration of sub-classification strategies in chronic non-specific LBP outcome research is needed. They proposed the development of explicit recommendations for the use of sub-classification strategies and evaluation of targeted interventions in future research evaluating chronic non-specific LBP [32].

Another systematic review [33] tried to determine the efficacy of targeted treatment for sub groups in adults with non-specific LBP. The results provide cautious evidence supporting the notion that treatment targeted to subgroups of patients with non-specific LBP may improve patient outcomes. However, the results of the studies included in this review are, inconsistent and the samples investigated are too small to make recommendations for clinical practice. The research suggests that adequately powered clinical trials using designs capable of providing robust information to support the modification of clinical practice are uncommon. Considering how central the notion of targeted treatment is to manual therapy principles, further studies using this research method should be a priority for the clinical and research communities.

A recent study emphasized stratification of management according to the patient's prognosis (low, medium, or high risk). They compared the clinical effectiveness and cost-effectiveness of stratified primary care (intervention) with non-stratified current best practice (control). 851 patients were assigned to the intervention (n = 568) and control groups (n = 283). Overall, adjusted mean changes in RMDQ scores were significantly higher in the intervention group than in the control group at 4 months and at 12 months. At 12 months, stratified care was associated with a mean increase in generic health benefit cost savings compared with the control group. The results suggest that a stratified approach, by use of prognostic screening with matched pathways, may have important implications for the future management of back pain in primary care[34].

#### Movement control

The importance of sub-classification has been highlighted in several studies. When sitting postures are compared between pain free subjects and patients with LBP, there are no significant differences [35-37]. However, when the patients are sub-classified into flexion and active extension control impairments, then the differences are significant. The direction of the movement control defines the way patients sit [37] and the activity in their back muscles; the flexion group has less back muscle activity and the active extension group more [36].

The MD is a clear subgroup of non-specific LBP. Pathokinesiologic movement patterns in the lumbar spine have been investigated and described [38-42].

Scholtes et al. [43] compared two groups of people who played rotation-related sports and their capability to control lumbar spine movement during knee flexion lateral hip rotation. The interpretation of that study was that patients with LPB have poorer control of their lumbopelvic movements, and because of this, might be moving in their daily activities and sports more on their lumbar spine which may cause pain. A significant difference in the ability to actively control the movements of low back between patients with low back pain and subjects without back pain has been demonstrated by Luomajoki et al. [44]. The effect size between patients with LBP and healthy controls in movement control is large.

The reliability of tests to diagnose MD has been shown to be acceptable. Dankaerts et al. [45] reported an almost perfect agreement (k = 0.96 and percentage agreement 97%) between two expert examiners rating a motor control dysfunction classification. Van Dillen et al. [46] used a battery of physical examination items in order to categorize the patients in an impairment dysfunction subgroup. They found a very high agreement for the assessment of symptoms among the examiners (k > 0.89 and percentage agreement > 98%). Luomajoki et al. [47] examined ten movement control tests for the back. Four blinded physiotherapists evaluated subjects through observation of videos. For the intraobserver reliability, five tests out of ten showed an excellent reliability (k > 0.80). Four further tests had a substantial reliability (k = 0.6-0.8) and one was moderate (0.51). Five out of ten tests showed a substantial inter-observer reliability (k > 0.6), four tests had Kappa values between 0.4 and 0.6 (good) and one test was under 0.4 (fair). The percentage agreement varied between 65% - 97.5%. White and Thomas [48] investigated the reliability (N = 37) of 16 tests of the Movement System Balance approach developed by Sahrmann [49], finding a satisfactory reliability between raters.

Harris-Hayes and van Dillen found overall percent agreement on the classification assigned to be 83% with kappa = 0.75 (95% confidence interval = 0.51-0.99; *P *< .0001) and concluded that inter-tester reliability of classification of patients with LBP when therapists use a standardized clinical examination based on the Movement System Impairment classification system is substantial [50]. Trudelle-Jackson et al. (2008) showed that interrater reliability between two physical therapists classifying patients with chronic LBP into 1 of 5 lumbar spine movement impairment categories had substantial agreement [51].

One recent study analysed the reproducibility of five different quantitative tests for those commonly used in daily clinical practice. These five tests for lumbar movement control displayed excellent reproducibility. There is no gold standard for movement control, therefore there are no diagnostic accuracy statistics available for these tests. The diagnostic accuracy of these tests needs to be addressed in larger cohorts of subjects, establishing values for the normal population. Also cut off points between subjects with and without LBP must be determined, taking into account age, level of activity, degree of impairment and participation in sports [52].

We are using the model presented by O'Sullivan [53]. In this classification of chronic LBP pain disorders, sub-groups, based on the mechanism underlying the disorder, are considered critical to ensure appropriate management. It is proposed that three broad sub-groups of chronic LBP disorders exist. In the first group of disorder patients present underlying pathological processes driving the pain, and the patients' motor responses in the disorder are adaptive. A second group disorders patients show psychological and/or social factors representing the primary mechanism underlying the disorder that centrally drives pain, and where the patient's coping and motor control strategies are mal-adaptive in nature. Finally it is proposed that there is a large group of chronic LBP disorders where patients present with either movement impairments (characterized by pain avoidance behaviour) or control impairments (characterized by pain provocation behaviour). These pain disorders are predominantly mechanically induced and patients typically undertake mal-adaptive primary physical and secondary cognitive compensations for their disorders that become a mechanism for ongoing pain. These subjects present either with an excess or deficit in spinal stability, which underlies their pain disorder[45].

#### Specific movement control exercises

The underlying hypothesis is that, due to poor MD of the back, the person is unknowingly damaging him- or herself through faulty movement patterns. O' Sullivan [53] describes these back pain patients not as pain avoiders, but, as pain provocateur. Relative flexibility theory [50] suggests that movement occurs through the pathway of least effort, e.g. if the hip movement is relatively stiff compared to that of the low back, then the movement is more likely to happen in the back, leading to a back pain problem related to the direction of that particular movement.

The directions or symptoms of the movement control are called flexion, extension and sideflexion/rotation.

To rehabilitate this type of MD, specific movement control exercises (SMCE) have been suggested [49]. These are exercises in which one joint (or region) is maintained in a neutral position with conscious control, either while an adjacent joint (or region) is independently moved, or while performing part of a functional movement, with normal breathing. The exercises require more sensory motor awareness and neurocognitive function to perform than general exercise [54]. They are generally performed with slow, low force repetitive movements. They can be performed with high load or with speed, however it is recommended that this is included in the description of the exercise protocol [Gibbons SGT, Newhook TW 2011 Specific movement pattern control exercise for low back pain: A systematic review. Submitted].

Evidence is gradually accumulating for the use of SMCE. A recent systematic review identified six randomized controlled trials, one prospective cohort study, one case control study, one case series and seven case studies that used SMCE. Based on four high quality RCT, the following levels of evidence were found: there is moderate evidence from one study for a long term (12 months) benefit of disability, pain and fear for the use of SMCE when combined with another form of active treatment and education for chronic LBP; there is moderate evidence from two studies for a short term (6 weeks) benefit of pain, pain interference and disability, for the use of SMCE when combined with active and passive treatments for chronic LBP; there is moderate evidence from one study for medium term (6 months) benefit of pain, disability and quality of life for the use of SMCE when combined with active and passive treatments for a mixed group of sub-acute and chronic LBP [Gibbons SGT, Newhook TW 2011 Specific movement pattern control exercise for low back pain: A systematic review. Submitted].

The clinical trials in this review used subjects with mostly chronic LBP. There is a need for knowledge of this type of intervention in sub-acute LBP.

### The aim of the study

The purpose of this study is to compare individually tailored SMCE to general exercise for reducing disability in patients with recurrent, sub-acute non-specific LBP after they have been sub-classified with MD.

## Methods/Design

The study protocol was registered on 18^th ^January 2012, registration number ISRCTN48684087 and approved by the Ethics Committee of Carea (Kymenlaakso Hospital District, Finland) in 17^th ^May 2010.

### Participants

#### Recruitment

Participants are 70 patients seeking treatment for sub-acute non-specific LBP from one physical therapy clinic in Kotka, Finland, between October 2010 and November 2012 (estimation).

#### Inclusion criteria

To be eligible for inclusion patients had to have non-specific LBP for at least 6 weeks, be aged between 16 and 65 years, and give written informed consent. Participants should have had at least one episode of LPB prior to the study. The aim of the rest inclusion regimen is to sub-classify those patients who have MD.

The participant should score Roland-Morris Disability Questionnaire [55] to be ≥ 5 points, DEPS [56] < 12 points, Tampa Scale for Kinesiophobia [57] < 38 points and Motor Control Abilities Questionnaire < 80 points. The Motor Control Abilities Questionnaire (MCAQ) is a self report tool that was developed to screen people for their ability to learn specific motor control stability exercise and specific movement control exercise. Reliability and validity have been established. A cut off point of 80 has a specificity of 0.98 and a sensitivity of 0.88 [58]. The MCAQ should be used to exclude those subjects who are unable to learn the exercises and thus not benefit from the treatment [59].

Within the physical examination the participant should have ≥ 2/6 positive MD test described by Luomajoki et al. [47]; should not have Straight Leg Raise (SLR) under 50° or any positive sacroiliac-joint pain provocation tests [60] to be eligible to participate in the study. Clinical assessment should indicate that the subject is suitable for active exercise, which is asked within a questionnaire.

#### Exclusion criteria

Potential participants are screened for evidence of serious low back pathology and for contraindications to exercise therapy by a physical therapist. They are excluded prior to randomization if they had neurological signs (leg weakness), specific spinal pathology (e.g. malignancy, or inflammatory joint or bone disease) or if they had undergone back surgery. The aim of the measurement of DEPS, TSK and MCAQ is to rule out those patients with LBP of non-mechanical origin, e.g. depression, fear-avoidance and a poor ability to learn exercises. The aim of physical examination of SLR and sacroiliac-joint provocation tests is to rule out those patients with mechanical movement impairment [60].

### Randomization

Each participant is randomized to a general exercise group or a SMCE group. Randomization will be done with the Randomizer 17.0 program. The randomization schedule is known only to one investigator who is not involved in recruiting participants, and it is concealed from patients and the other investigators using consecutively numbered, sealed, opaque envelopes. The physiotherapists treating the participants are not involved in the randomization process.

Baseline assessment of each group will be taken to ensure they are not different.

### Interventions

Participants attend for up to five treatment sessions over an eight week period. The treatment is carried out by two different physiotherapists. The treatments are implemented as follows.

### Initial assessment

A physical therapist carries out an initial assessment of each participant allocated to the exercise group to determine how physically active the participant is, how troublesome the back problem is, and the ability of the participant to perform the exercises. These are measured by the treating physiotherapist by asking the participant.

#### General exercise

Participants are taught the exercises and advised of the intensity at which they should exercise. The exercises are performed under supervision of a physical therapist. The intensity of the exercises is progressed over the 5 treatments with participants being encouraged to improve their own performance. Each session lasts 45 min and includes a short session (10-15 minutes) of manual therapy. Home exercises are taught and the ability to perform them is controlled in each treatment session. The participant performs the previously taught exercises and the physiotherapist corrects the performance if necessary. Home exercises are instructed to be performed three times a week.

The main aims of the program are to improve physical function and confidence in using the spine. The program targeting at abdominal and paraspinal muscles without the involvement of the deep muscles activation was described by McGill [22] and was investigated by Koumantakis et al. (2005) [19].

#### Specific movement control exercise

Participants are taught the SMCE and advise of the intensity at which they should exercise. The exercises are performed under supervision of a physical therapist. The participant performs the previously taught exercises and the physiotherapist corrects the performance if necessary. In addition the movement control is taught with sitting position exercises, four point kneeling and standing exercises according to the decision of the physical therapist to be performed once or twice daily. The intensity of the exercises is progressed over the 5 treatments with participants being encouraged to improve their own performance. Each session lasts 45 min and includes a short session (10-15 minutes) of manual therapy. Home exercises are taught and the ability to perform them is controlled in each treatment session. Home exercises are taught to be performed three times a week and the sitting, four point kneeling and standing exercises are taught to be performed once or twice daily.

The main aims of the program are to improve the individual direction specific movement control of the lumbar spine, physical function and confidence in using the spine.

**Figure 1:**
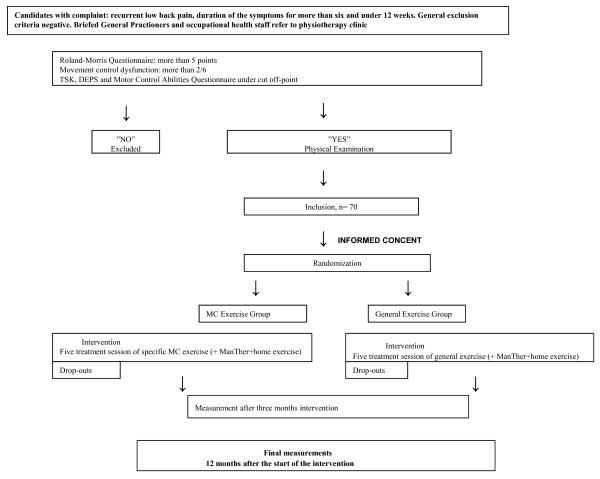
**Flow chart**. LBP: low back pain; RMDQ: Roland-Morris Disability Questionnaire; MD: movement control dysfunction; TSK: Tampa Scale for Kinesiophobia; DEPS: a depression questionnaire; MCAQ: Motor Control AbilitiesQuestionnaire; SMQE: specific movement control exercise; MC: movement control: ManTher: manual therapy.

The main difference between the two exercise groups is individual and also cognitive learning, because in SMCE group the participants also learn how to move and use their back Figure 1.

### Outcome measures

Baseline measures are taken of the one primary outcome (RMDQ), and four secondary outcomes (PSFS and Oswestry Disability Index, Movement control tests by Luomajoki, general health questions) prior to randomization.

Main outcome measure:

Roland-Morris Disability Questionnaire (RMDQ)

Secondary outcome measures:

Patient-Specific Functional Scale (PSFS) [61]

Oswestry Disability Index [62]

Movement control tests described by Luomajoki et al. [47]

The amount of absence from work with a questionnaire#

The need for other treatment modalities with a questionnaire#

The need for pain medication with a questionnaire#

Patient satisfactory with global assessment with a questionnaire#

# A questionnaire with three claims (less than usual, equal, more than usual)

### Statistical analysis

A sample size of 70 participants, determined a priori, provides 80% power by α 0.05 to detect an effect of change in disability based on three point difference with RMDQ, which we regard as minimal important difference for this outcome [63].

The comparability of the groups on prognostic and outcome variables at baseline will be analyzed using the two-sample t-tests for parametric and Wilcoxon test for non-parametric distribution as well as Chi-Square test for nominal data. Differences between the groups over time are measured with Mann Whitney *U *test. A regression analysis for predictive factors will be conducted on covariates at baseline. Statistical significance is set on α < 0.05. Statistical analyses will be done with SPSS for Windows release 17.0.

## Discussion

The aim of this study is to compare SMCE and general exercises within the sub-group of patients with MD in the sub-acute stage of LBP. The main study question is which of the two exercise programs is more effective in reducing the disability associated with LBP.

European clinical guideline for management of chronic LBP recommends that more research is required to develop tools to improve the classification and identification of specific clinical sub-groups of chronic LBP patients [5]. Good quality RCTs are then needed to determine the effectiveness of specific interventions aimed at these specific risk/target groups. There is a need to evaluate sub-acute non-specific LBP and need to study interventions aimed at subgroup of patients with MD.

This RCT aims to test the hypothesis whether patients within a sub-group of MD benefit more through a specific individually tailored exercise program than through general exercise.

The participants in this trial are unique population; a sub-classification of MD or movement control impairment in non-specific LBP patients will show that the findings of this trial can confidently be applied to similar populations. The comparison is between two exercise programs and therefore the data should not be used to make inferences about the effectiveness, compared to no intervention, of any of the treatments. The findings can assist care providers, therapists and people with sub-acute LBP to make rational decisions about treatment. Care providers will need to take into account how the interventions are administered.

The study protocol of investigating patients with sub-acute LBP is important. If there are effective ways of preventing LBP to become chronic, the high costs of treating patients could be avoided.

This study has several limitations. The treating physiotherapist or subjects cannot be blinded, however because there is no accepted standard therapy, it is not truly known which therapy is better. The amount of the home exercises is totally dependent the motivation of the subject to perform the given exercise program which could influence the outcome of this study. Core stability represents a spectrum of exercises [64]. The comparison group includes a group of core stability exercises, core stiffness exercises, that involves an element of control of the spine. This means that both groups have an intervention that is cognitively attempting to control the position of the spine, although they also have fundamental differences in their application and potential benefits. This study includes subjects with recurrent, sub-acute LBP. Some of these individuals may spontaneously recover [4]. With a small sample size, the results would have to be interpreted with caution. There are several aspects of the study which influence the external validity. The application of the interventions within the study relies on the skills of the treating physiotherapist. Physiotherapists could learn to teach general exercise program, but the assessment and rehabilitation of MD is not taught in all undergraduate courses and post graduate training is required. This study will use five treatment sessions, however it may take longer for some patients to learn the SMCE well enough to change the movement patterns and decrease disability. In practice, greater than five sessions is likely possible, however if the SMCE are not effective in reducing disability, it is not known what would happen with a longer rehabilitation time frame with more sessions. In clinical practice, time and costs often limit the time that a physiotherapist can spend with a patient. It may not be appropriate to physiotherapists to spend forty-five minutes with patients as was done in this study. This could influence the application of learning the SMCE.

The main study question is which of the two exercise programs is more effective in reducing the disability associated with LBP.

## Competing interests

The authors declare that they have no competing interests.

## Authors' contributions

VL and HL are responsible for the design of the study. VL and OA helped with the methodological considerations and Gibbons helped with the study design, methodological considerations and text editing. All authors have read and approved the final manuscript.

## Pre-publication history

The pre-publication history for this paper can be accessed here:

http://www.biomedcentral.com/1471-2474/13/55/prepub
